# Design, Synthesis, and Molecular Docking Study of Novel Heterocycles Incorporating 1,3,4-Thiadiazole Moiety as Potential Antimicrobial and Anticancer Agents

**DOI:** 10.3390/molecules24061066

**Published:** 2019-03-18

**Authors:** Mohamed El-Naggar, Hanan A. Sallam, Safaa S. Shaban, Salwa S. Abdel-Wahab, Abd El-Galil E. Amr, Mohammad E. Azab, Eman S. Nossier, Mohamed A. Al-Omar

**Affiliations:** 1 Chemistry Department, Faculty of Sciences, University of Sharjah, Sharjah 27272, UAE; m5elnaggar@yahoo.com; 2 Synthetic Organic Laboratory, Chemistry Department, Faculty of Science, Ain Shams University, Cairo 11566, Egypt; dr.salam.h.a@gmail.com (H.A.S.); safashaban@ymail.com (S.S.S.); meazabali2015@yahoo.com (M.E.A.); 3 Faculty of Pharmaceutical Sciences and Pharmaceutical Industries, Future University in Egypt, New Cairo 11835, Egypt; salwa_elsayed@ymail.com; 4 Pharmaceutical Chemistry Department, Drug Exploration and Development Chair (DEDC), College of Pharmacy, King Saud University, Riyadh 11451, Saudi Arabia; malomar1@ksu.edu.sa; 5 Applied Organic Chemistry Department, National Research Centre, Cairo 12622, Egypt; 6 Pharmaceutical Medicinal Chemistry Department, Faculty of Pharmacy (Girls), Al-Azhar University, Cairo 11754, Egypt; dr.emannossier@gmail.com

**Keywords:** 1,3,4-thiadiazole, antimicrobial, anticancer, dihydrofolate reductase, molecular docking

## Abstract

A new series of 5-(3,5-dinitrophenyl)-1,3,4-thiadiazole derivatives were prepared and evaluated for their in vitro antimicrobial, antitumor, and DHFR inhibition activity. Compounds **9**, **10**, **13**, and **16** showed strong and broad-spectrum antimicrobial activity comparable to Amoxicillin and Fluconazole as positive antibiotic and antifungal controls, respectively. Compounds **6**, **14**, and **15** exhibited antitumor activity against four human cancer cell lines, CCRF-CEM leukemia, HCT-15 colon, PC-3 prostate, and UACC-257 melanoma cell lines using Doxorubicin as a reference drug. Compounds **10**, **13**, **14**, and **15** proved to be the most active DHFR inhibitors with an IC_50_ range of 0.04 ± 0.82–1.00 ± 0.85 µM, in comparison with Methotrexate (IC_50_ = 0.14 ± 1.38 µM). The highly potent DHFR inhibitors shared a similar molecular docking mode and made a critical hydrogen bond and arene‒arene interactions via Ser59 and Phe31 amino acid residues, respectively.

## 1. Introduction

Dihydrofolate reductase (DHFR) is a prevalent enzyme that is present in all prokaryotic and eukaryotic cells. It has a key role in folate metabolism and subsequently DNA and RNA synthesis [1,2]. Inhibition of DHFR revealed importance in the development of therapeutic agents against anticancer drugs as well as bacterial and parasitic infections [3,4,5,6,7]. DHFR inhibitors exhibit a vital role in clinical medicine, like the use of Methotrexate in neoplastic diseases, inflammatory bowel diseases, rheumatoid arthritis, psoriasis, and asthma [8].

**Figure 1:**
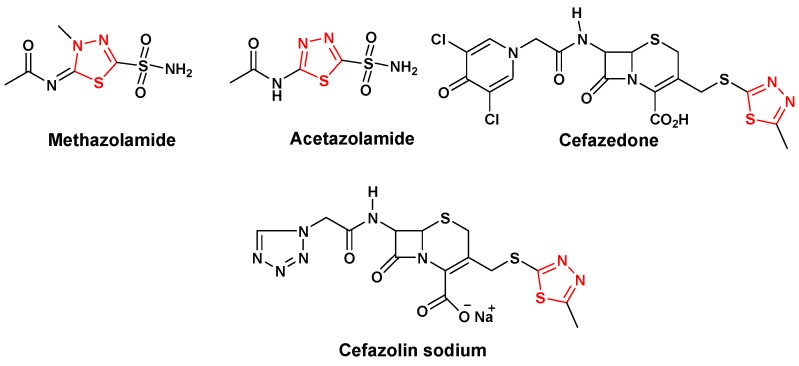
Some drugs on the market possessing a 1,3,4-thiadiazole scaffold.

1,3,4-Thiadiazole is a privileged five-membered ring system that has gained prominence by exploring broad biological activity spectrum due to the presence of the N=C‒S moiety. From the literature survey, it was noticed that 1,3,4-thiadiazole derivatives possess many pharmacological activities, such as antimicrobial, anti-hepatitis B viral, antitubercular, antileishmanial, analgesic, anti-inflammatory, anticancer, anticonvulsant, central nervous system (CNS) depressant, antioxidant, molluscicidal, antidiabetic, diuretic, and antihypertensive activities [9,10,11,12,13,14,15,16,17,18]. Furthermore, many drugs containing a 1,3,4-thiadiazole nucleus have been approved, such as methazolamide, which is used as a carbonic anhydrase inhibitor, and acetazolamide, which is a modulator of anticancer therapies in combination with different cytotoxic agents [19,20]. Other thiadiazole-containing drugs include Cefazedone and Cefazolin sodium, which have broad-spectrum antibiotic action [21] (Figure 1).

Recently, some pharmacophores containing 1,3,4-thiadiazoles have been explored for their potential antitumor and antimicrobial activities via inhibition of DHFR. For example, 2-hydrazono-1,3,4-thiadiazole **I** was reported to possess promising antitumor and antimicrobial activities [22]. Moreover, compound **II** belongs to the 3-(4-nitrophenyl)-5-(thiophen-2-yl)-2,3-dihydro-1,3,4-thiadiazole skeleton, has high anticancer potency comparable to Cisplatin, and was allocated with DHFR inhibition [23].

**Figure 2:**
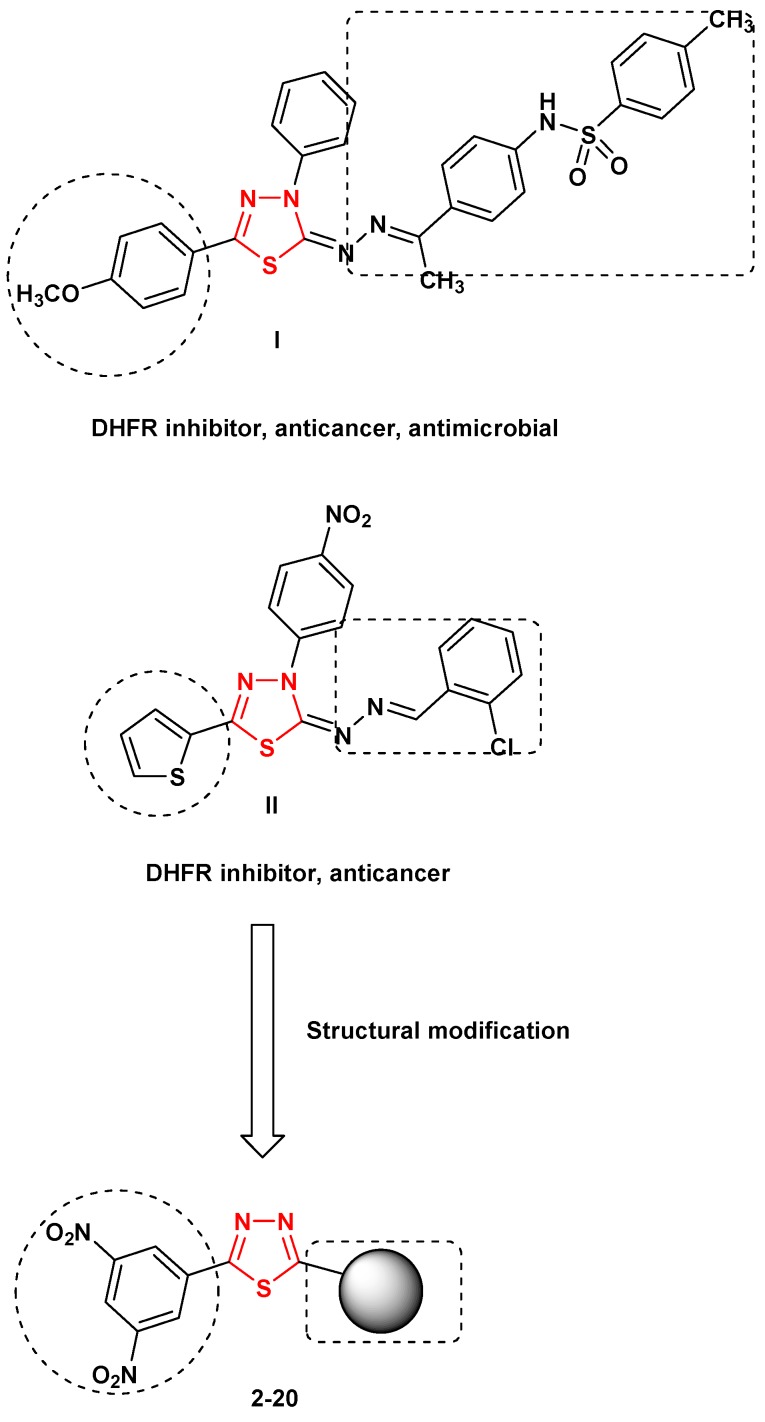
Structures of previously reported 1,3,4-thiadiazoles **I**, **II** with DHFR inhibitory activity and the strategy employed for designing the lead compounds **2**–**20**.

Considering the findings above and as a continuation of our efforts towards the development of biologically active heterocyclic compounds [24,25,26,27,28,29,30,31,32,33,34], we undertook the design and synthesis of some novel 1,3,4-thiadiazole prototypes that possess the advantages of pharmacophores (**I**, **II**), as outlined in Figure 2. All the newly hybrid compounds comprising the 1,3,4-thiadiazole motif were evaluated for their antimicrobial and anticancer activities through a study of their in vitro inhibitory activity enzyme against DHFR enzyme, followed by molecular docking studies to get insight into the interactions and binding modes in the active site of this enzyme.

## 2. Results and Discussion

### 2.1. Chemistry

In this study, compound 5-(3,5-dinitrophenyl)-1,3,4-thiadiazol-2-amine (**1**) was used as the key starting material to synthesize different heterocyclic compounds.

Thus, when compound **1** was treated with carbon disulfide and potassium hydroxide, followed by stirring with hydrazine hydrate, thiosemicarbazide derivative **2** was produced.

Compound **2** was used as a precursor to construct different heterocyclic ring systems such as thiadiazole, thiazole, and pyrimidine through the reaction with different reagents.

**Scheme 1:**
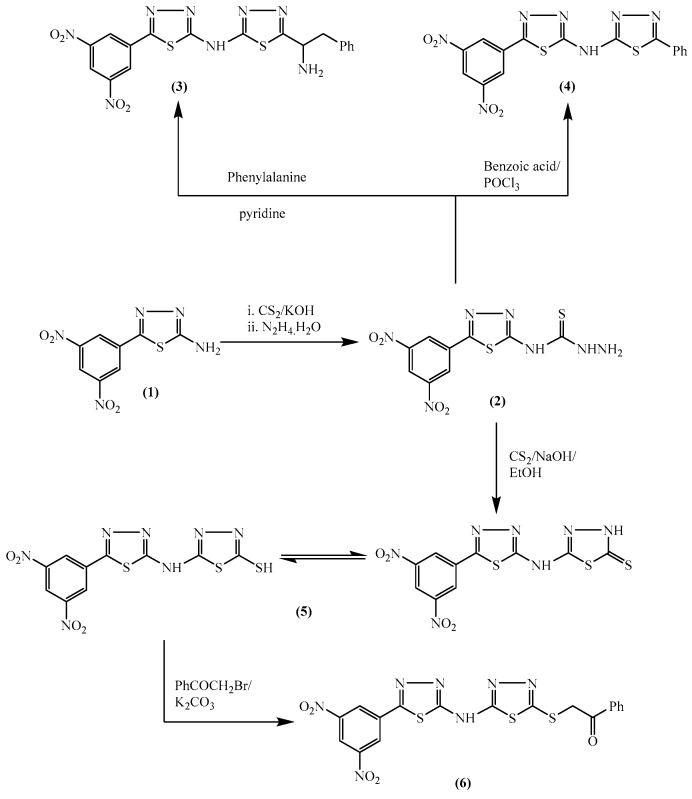
Synthesis route for compounds **2**–**6**.

Consequently, compound **2** was treated with phenylalanine/pyridine, benzoic acid/POCl_3_, and/or CS_2_/NaOH to give 1,3,4-thiadiazol-2-yl-1,3,4-thiadiazole derivatives **3**–**5** in a good yield. Reacting compound **5** with phenacyl bromide in the presence of potassium carbonate produced the acetophenone derivative **6** (Scheme 1).

Compound **3** was formed via a nucleophilic attack of the NH_2_ group of thiosemicarbazide on the carbonyl group of phenylalanine through tetrahedral mechanism, followed by elimination of the water molecule; then ring closure takes place via the elimination of another water molecule from the thiol and OH of the carboxylic group. However, in the case of compound **4** the carboxylic group is converted into acid chloride and as a result a thiadiazole ring is formed by the elimination of water and HCl molecules. In compound **5**, a nucleophilic attack of NH_2_ group of thiosemicarbazide on the C=S group was followed by ring closure through the elimination of the H_2_S molecule.

The structures of the products were in accordance with their elemental analysis and spectral data, where the IR spectrum of compound **3** showed bands at 3415-3365, 3257, 2958, and 2924, for the NH_2_, NH, CH_2_, and CH groups, respectively. Its ^1^H-NMR spectrum exhibited peaks at δ 3.17, 3.31 (m, 2H, CH_2_-Ph), 4.08 (t, 1H, CH), 6.45 (s, 2H, NH_2_), and 11.21 (s, 1H, NH). 

As proof of the proposed structure of compound **4**, its IR spectrum displayed a stretching frequency of 3265 (NH) and the 1H-NMR spectrum had peaks at δ 7.33–7.66 (m, 5H, Ph-H) and 10.22 (s, 1H, NH). For compound **5**, the IR spectrum showed two characteristic absorption bands at 3261 (NH) and 2561 (SH), which indicates the presence of the compound in the thiol form, besides the 1H-NMR peaks at δ 10.13 and 12.44 corresponding to the NH and SH groups. The IR spectrum of compound **6** lacked the band of the SH group and showed bands at 3269 (NH) and 2921 (CH_aliph_.), and the ^1^H-NMR spectrum displayed peaks at δ 4.96 (s, 2H, S‒CH_2_‒CO), 7.37–7.69 (m, 5H, Ph-H) and 10.29 (s, 1H, NH).

**Scheme 2:**
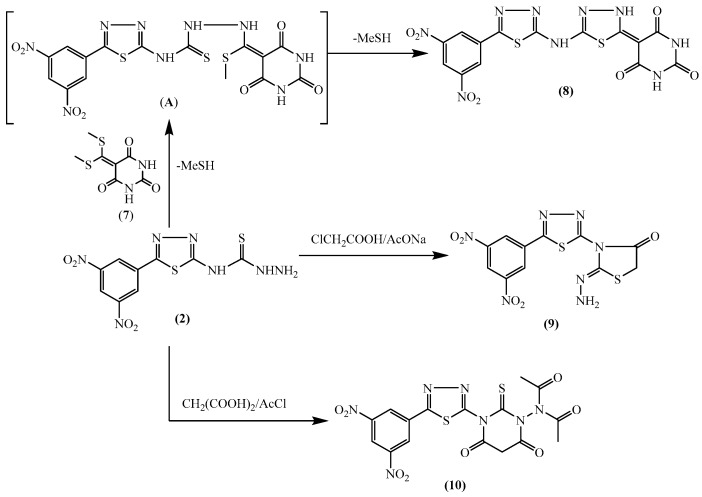
Synthesis route for compounds **8**–**10**.

Furthermore, thiosemicarbazide **2** reacted with bis-methylthiomethylene-barbituric acid (**7**), chloroacetic acid/sodium acetate, and/or malonic acid in the presence of acetyl chloride to give barbituric, thiazolidin-4-one, and thiobarbituric derivatives **8**–**10**, respectively (Scheme 2).

The mechanism for the formation of compound **8** could be as follows: first, an Michael addition reaction between the NH_2_ of the thiosemicarbazide **2** and the bis-methyl-thiomethylene barbituric acid (**7**), resulting in the elimination of the CH_3_SH group to give intermediate (A), which underwent intramolecular cyclization through the elimination of the second CH_3_SH group to furnish the pyrimidine-2,4–6-trione derivative **8**.

The structure of compound **8** was elucidated from its elemental analysis and spectral data. The IR spectrum revealed the following bands: 3322–3189 (NH groups) and 1744, 1678, and 1655 (C=O groups). The ^1^H-NMR spectrum exhibited four peaks at δ 9.34, 11.19, 12.67, and 12.78 (4s, 4H, 4NH).

Compound **9** could be formed via nucleophilic attack of the nitrogen atom of thiosemicarbazide on the carbonyl group of chloroacetic acid with elimination of the H_2_O molecule; then ring closure takes place through nucleophilic attack of the sulfur atom (in the thiol form) on the carbon atom (attached to Cl), synchronized with elimination of the HCl molecule.

The spectral data were in accordance with the proposed structure of compound **9**, where the IR spectrum showed characteristic stretching frequencies at 3378, 3254 (NH_2_), and 1681 (C=O), while the ^1^HNMR spectra displayed peaks at δ 3.86 (s, 2H, C5 thiazolidine) and 6.41 (s, 2H, NH_2_).

The attack of two NH groups of thiosemicarbazide on the two carbonyl groups of malonic acid resulted in ring closure with elimination of two water molecules to produce compound **10**, accompanied by acylation of the NH_2_ group.

**Scheme 3:**
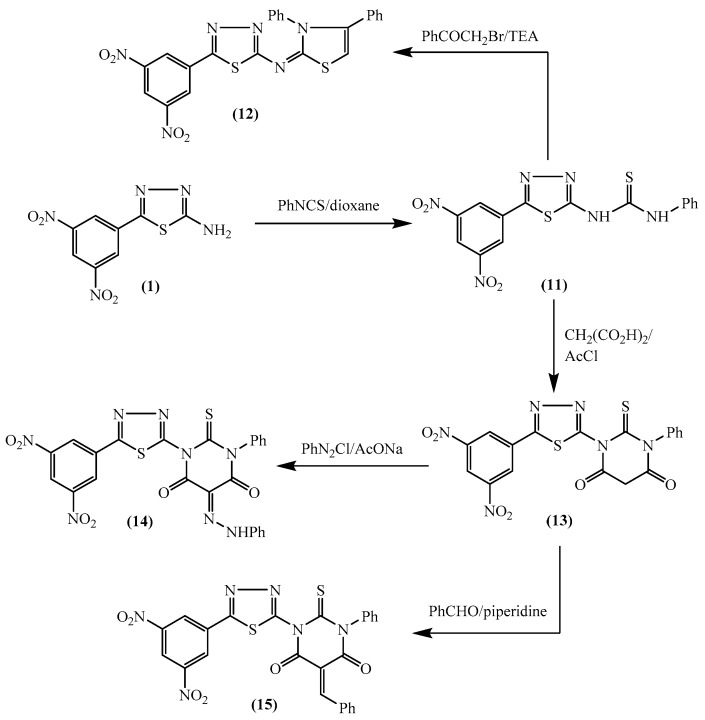
Synthesis route for compounds **11**–**15**.

Meanwhile, compound **1** was allowed to react with phenylisothiocyante in dioxane to produce the thiadiazole derivative **11**, which may be formed as a result of the nucleophilic attack of the NH_2_ on the carbon atom of the isothiocyant derivative (Scheme 3).

Compound **11** was supported by its spectral data: its IR spectrum displayed stretching absorption bands at 3329 and 3256, corresponding to two NH groups. At the same time, ^1^H-NMR revealed peaks at δ 10.49, 11.33 (2s, 2H, 2NH), and δ 7.45–7.89 (m, 5H, Ar-H).

The reactivity of compound **11** was explored through the reaction with phenacyl bromide in the presence of triethylamine as a catalyst, and/or malonic acid/acetyl chloride to give thiazole and thiobarbituric derivatives **12** and **13**, respectively (Scheme 3).

The formation of compound **12** could be via a nucleophilic attack of NH on the carbonyl group, resulting in the elimination of a water molecule; then, the sulfur in the thiol form attacks the methylene group, leading to ring closure, accompanied by elimination of HBr molecule to produce the thiazole derivative **12**.

The spectral data of compound **13** showed the following bands in the IR spectrum: 2961 (CH_aliph_), and 1702, 1688 (C=O), and ^1^H-NMR displayed peaks at δ 3.31 (s, 2H, C5 pyrimidine) and 7.32–7.88 (m, 5H, Ar‒H).

Compound **13** was coupled with benzene diazonium chloride in the presence of sodium acetate and/or condensation with benzaldehyde in the presence of piperidine to produce phenyl hydrazone and thiadiazole derivatives **14** and **15**, respectively (Scheme 3).

The IR spectrum of **14** exhibited a stretching frequency band at 3276, corresponding to the NH group, and its ^1^H-NMR spectrum revealed peaks at δ 7.25–7.69 (m, 10H, Ar-H) and 12.55 (s, 1H, NH), while the ^1^H-NMR of **15** showed peaks at δ 6.84 (s,1H, olefinic proton) and 7.41–7.77 (m, 10H, Ar-H).

The present investigation was extended to demonstrate the reactivity of dimethyl carbon-imidodithioate derivative **16** towards some amine derivatives in order to synthesize different heterocyclic systems. Compound **16** was prepared through the treatment of the 2-aminothiadiazole derivative **1** with carbon disulfide in a basic medium followed by S-methylation using methyl iodide.

**Scheme 4:**
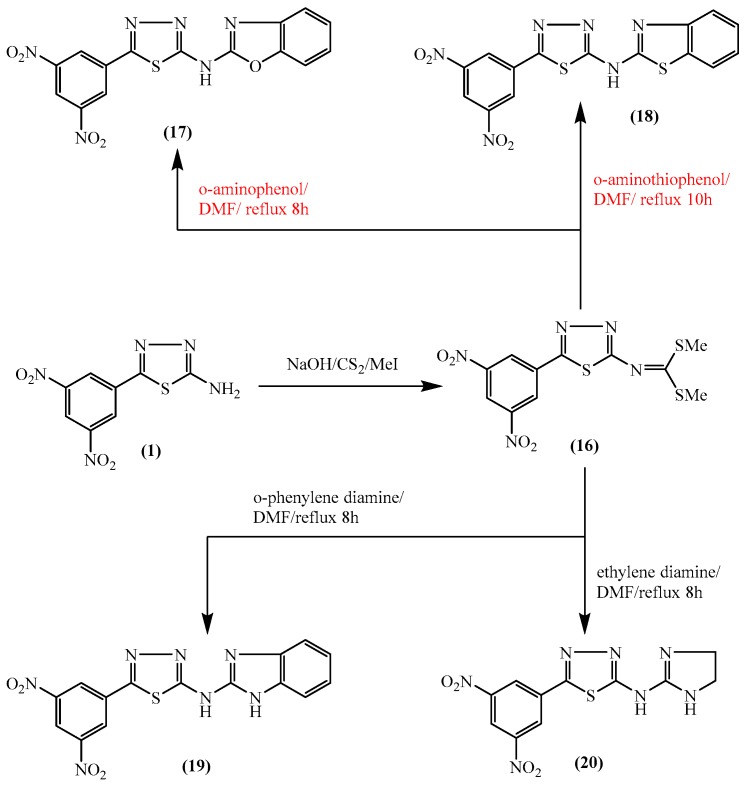
Synthesis route for compounds **16**–**20**.

When compound **16** was allowed to react with amines, namely o-aminophenol, o-amino-thiophenol, o-phenylenediamine, and/or ethylene diamine in dimethylformamide, benzo-oxazole, benzothiazole, benzoimidazole, and dihydroimidazole derivatives **17**–**20** were produced, respectively (Scheme 4).

**Scheme 5:**
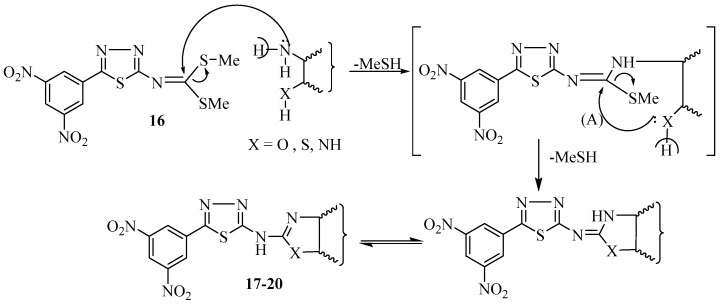
Speculated mechanism for the formation of compounds **17**–**20**.

The formation of compounds **17**–**20** was proposed to take place via the nucleophilic attack of the amino and HX groups on the carbon atom of thioacetal followed by elimination of two MeSH molecules. This can be shown as a speculated mechanistic pathway in Scheme 5.

The elemental analyses and spectral data were in accordance with the proposed structures of compounds **17**–**20**. The IR spectra showed stretching absorption bands at 3389–3272 for NH groups. Also, the ^1^H-NMR spectra of compounds **17**–**19** showed peaks at δ 7.17–7.51 (m, 4H, Ar-H), 9.78 (s, 1H, NH), 7.36–7.67 (m, 4H, Ar-H), 11.06 (s, 1H, NH), 7.11–7.43 (m, 4H, Ar-H), 9.96 (s, 1H, NH) and 11.78 (s, 1H, NH), respectively, while the ^1^H-NMR spectrum of compound **20** exhibited peaks at δ 2.81 (t, 2H, CH_2_-imidazole), 2.69 (t, 2H, CH_2_-imidazole), 9.83 (s, 1H, NH), and 10.66 (s, 1H, NH), respectively.

### 2.2. Biological Evaluation

#### 2.2.1. Antimicrobial Sensitivity Assay

**Figure 3:**
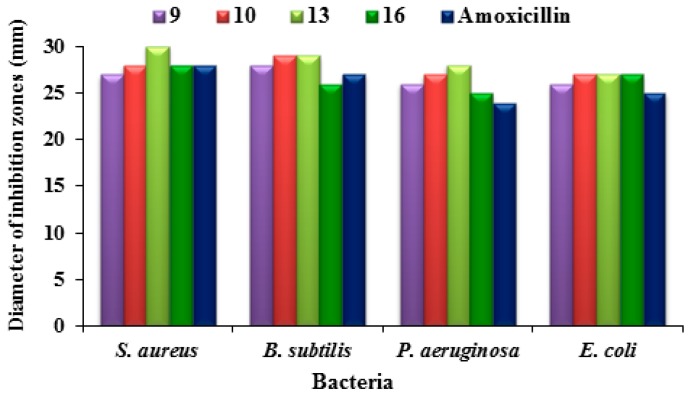
Antimicrobial activity of the most potent compounds against different bacterial strains.

**Figure 4:**
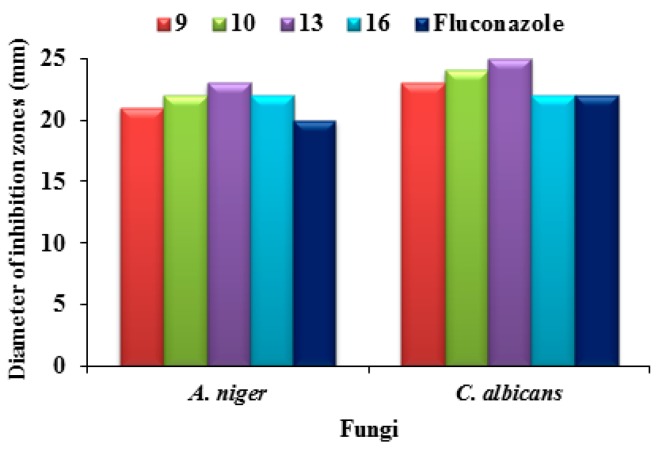
Antifungal activity of the most potent compounds against different fungal strains.

All the synthesized compounds **1**–**20** were screened for their antimicrobial activity against Gram-positive bacteria (*Staphylococcus aureus* and *Bacillus subtilis*), Gram-negative bacteria (*Pseudomonas aeruginosa* and *Escherichia coli*), and yeast-like pathogenic fungi (*Aspergillus niger* and *Candida albicans*). The antimicrobial screening was carried out using a standard well agar diffusion assay according to Cheesbrough et al. [35]. The broad-spectrum antibiotic Amoxicillin and the antifungal Fluconazole were used at a concentration of 100 μg/mL as positive controls. The obtained results (Table 1) revealed that the tested compounds **9**, **10**, **13**, and **16** showed the highest antimicrobial activity. Compound **13** is the most potent one since it demonstrated antimicrobial activity higher than that of the standard drugs (Figure 3 and Figure 4). The rest of the tested compounds had antimicrobial activity that ranged from moderate to weak.

#### 2.2.2. Structure–Activity Relationship for Antimicrobial Activity

By analysis of the previous results and concerning the structural modifications that occurred only at position-2 (p-2) of the parent 1,3,4-thiadiazole scaffold, it was found that: a 1,3,4-Thiadiazole derivative **1** having free amino group at p-2 showed moderate inhibitory activity against all tested strains. Substitution of NH_2_ group with thiocarbohydrazide in compound **2** produced excellent activity, while its phenyl congener **11** displayed moderate activity. Furthermore, substitution of the amino group with dimethyl carbonimidodithioate in compound **16** exhibited high inhibitory activity and was approximately equipotent with the reference.

The existence of a new 1,3,4-thiadiazole ring via the NH linker revealed various levels of antimicrobial activity:

- a drop in the potency, illustrated in substitution at p-2 of the new 1,3,4-thiadiazole moiety with 1-amino-2-phenylethyl in compound **3** or with phenyl in compound **4**;
- elevated potency if the same position in the new 1,3,4-thiadiazole moiety is substituted with a free thioxo group in compound **5**; and
- moderate activity but only about half that of compound **5** if the thioxo group is substituted with phenylethane-1-one in compound **6**, or p-2 is linked to pyrimidine-2,4,6-trione scaffold in compound **8**.

Direct attachment of the thiazole moiety achieved excellent potency in compound **9**, while attachment via an imine group led to a drop in activity in compound **12**. 

Moreover, direct attachment of a thioxopyrimidine-4,6-dione scaffold led to excellent activity with substitution at N-1 either with phenyl as in **13** or with acetyl acetamide as in **10**. It was noted that insertion of 2-phenyl hydrazone in compound **14** or benzylidine in compound **15**, to p-5 of the newly formed 2-thioxopyrimidine-4,6-dione of **10** and **13**, decreased the activity to a moderate level.

Upon attachment of an imidazole moiety via a NH linker there was significant antimicrobial activity in compound **20**, followed by a drastic drop due to cyclization forming benzo[*d*]imidazole derivative **19** and its congeners benzo[*d*]oxazole and benzo[*d*]thiazole derivatives **17**, **18**, respectively.

#### 2.2.3. In Vitro Anticancer Activity

All synthesized compounds **1**–**20** were investigated for their in vitro cytotoxic activity against four human carcinoma cell lines, CCRF-CEM, HCT-15, PC-3, and UACC-257, using Doxorubicin as a standard anticancer drug. The anticancer activity was expressed as IC_50_ values (the concentration of test compounds required to kill 50% of the cell population) in (μM) ± SEM from three replicates.

The results depicted in Table 2 revealed that the most potent compounds are **15** > **14** > **6**, in descending order, against all tested cell lines. Compounds **8** and **12** exhibited moderate activity about half that of the reference drug. The rest of the tested compounds showed weak cytotoxic activity.

Comparing the IC_50_ values obtained for the synthesized derivatives against CCRF-CEM, HCT-15, PC-3, and UACC-257 with those obtained against non-tumorigenic MCF-10A cells, we can conclude that the synthesized derivatives have much less toxicity against normal cells.

#### 2.2.4. Structure–Activity Relationship for Anticancer Activity

Regarding the chemical structural modifications at p-2 of the parent 1,3,4-thiadiazole moiety, it was observed that: incorporation of 2-thioxopyrimidine-4,6-dione having benzylidene group at p-5 in compound **15** gave the highest cytotoxic activity (IC_50_ = 6.99 ± 0.4, 5.28 ± 0.5, 4.67 ± 0.3 and 7.41 ± 0.5 μM, respectively), which is equipotent to the reference drug (IC_50_ = 6.78 ± 0.7, 5.17 ± 0.2, 4.56 ± 0.4 and 7.34 ± 0.5 μM, respectively). Alteration of benzylidene with 2-phenyl hydrazone in compound **14** maintained high potency but slightly less than compound **15** (IC_50_ = 9.66 ± 0.6, 8.59 ± 0.7, 6.99 ± 1.2 and 9.20 ± 0.8 μM, respectively).

Also, increased potency was obtained upon addition of a new 1,3,4-thiadiazole ring substituted with thio-1-phenylethan-1-one group via a NH linker in compound **6** (IC_50_ = 10.99 ± 0.3, 9.87 ± 0.5, 9.92 ± 1.2 and 12.67 ± 0.6 μM, respectively).

On the other hand, moderate activity was caused by the attachment of new 1,3,4-thiadiazole ring bearing pyrimidine-2,4,6-trione group via a NH linker in compound **8** (IC_50_ = 19.19 ± 0.5, 17.24 ± 0.9, 15.44 ± 1.4 and 22.66 ± 1.3 μM, respectively) or by insertion of a 3,4-diphenylthiazole scaffold via an imine linker in compound **12** (IC_50_ = 15.78 ± 0.8, 15.01 ± 0.5, 12.14 ± 1.1 and 18.38 ± 1.6 μM, respectively).

#### 2.2.5. Dihydrofolate Reductase (DHFR) Inhibition

The synthesized compounds **1**–**20** were evaluated as inhibitors of bovine liver DHFR using a reported procedure [36]. Results were summarized as IC_50_ values in Table 3 and Methotrexate was used as a positive control. As illustrated in Table 3, compounds **10**, **13**, **14**, and **15** proved to be the most active inhibitors with an IC_50_ range from 0.04 ± 0.82 to 1.00 ± 0.85 µM, in comparison with Methotrexate (IC_50_ = 0.14 ± 1.38 µM). However, the rest of the tested compounds showed moderate to weak inhibitory activity, with an IC_50_ range of 8.46 ± 0.13–36.48 ± 0.72 µM.

The inhibitory activity of the tested derivatives could be correlated to structure variation and modifications. By investigating the variation in selectivity of the highly potent compounds **10**, **13**, **14**, and **15** over the DHFR enzyme, it was revealed that the existence of thioxopyrimidine-4,6-dione moiety at p-2 of the parent 1,3,4-thiadiazole scaffold led to enhanced activity, and the potency order was **15** > **14** > **13** > **10**. Structure–activity relationships in these compounds demonstrated that compounds with substitution at p-5 of the newly formed 2-thioxopyrimidine-4,6-dione (**14**, **15**, with excellent anticancer activity) showed more potent inhibitory activity against the DHFR enzyme than those having no substituents (**10**, **13**, with excellent antimicrobial activity).

### 2.3. Molecular Modeling Studies

**Figure 5:**
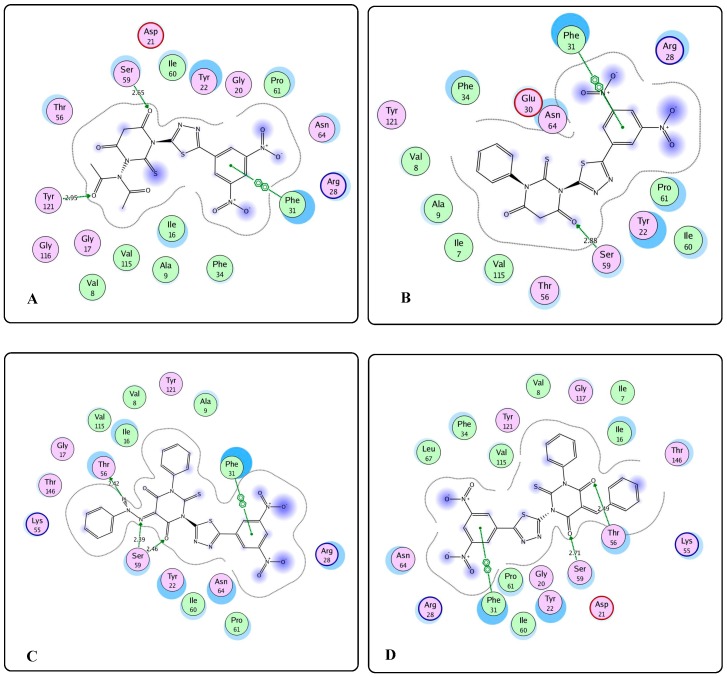
Two-dimensional representation of the interactions of compounds **10** (**A**), **13** (**B**), **14** (**C**), and **15** (**D**) with the DHFR enzyme pocket amino acids.

**Figure 6:**
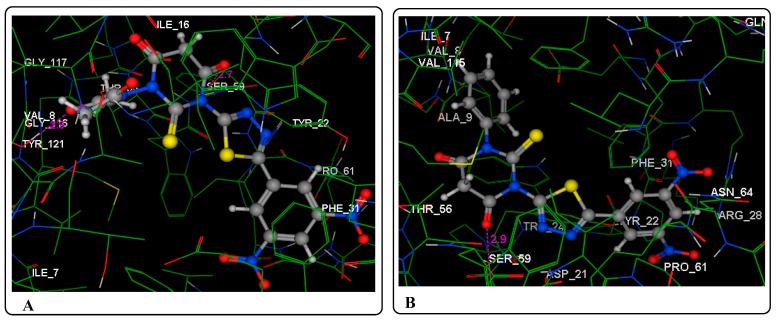

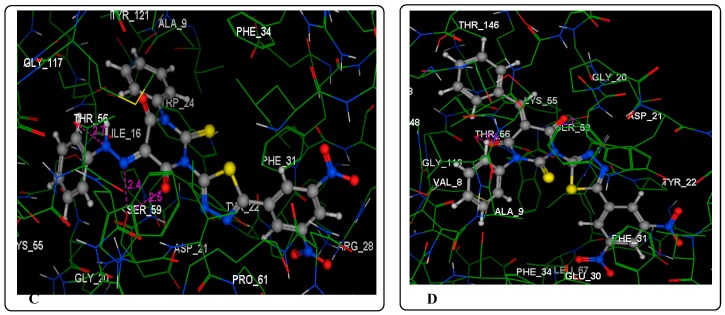
Three-dimensional representation of the interactions of compounds **10** (**A**), **13** (**B**), **14** (**C**), and **15** (**D**) with the DHFR enzyme pocket amino acids.

To gain a better understanding of the potency of the studied compounds and guide further SAR studies, we proceeded to examine the interaction of compounds **10**, **13**, **14**, and **15** via the X-ray crystallographic structure of DHFR (PDB ID: 1DLS) [37]. The co-crystallized ligand Methotrexate was redocked into the pocket sites of DHFR and revealed docking score energies of −11.4 kcal/mol at a root mean square deviation (RMDS) value of 9.1. The molecular docking was performed by inserting compounds **10**, **13**, **14**, and **15** into the ATP binding site of DHFR. All docking runs applied the LigandFit Dock protocol of Molecular Operating Environment (MOE, 10.2008) software [38,39]. The docking scores for compounds **10**, **13**, **14**, and **15** were all in the range −15.6 to −12.3 kcal/mol. Representations of the docking results of these compounds and DHFR are given in Figure 5 and Figure 6.

Inspection of the binding modes demonstrated that all compounds were potently bound to the ATP binding site of DHFR via arene‒arene interaction between the centroids of Phe31 and 3,5-dinitrophenyl moiety, and a hydrogen bond acceptor between the sidechain of Ser59 and one oxygen of thioxopyrimidine-4,6-dione.

Furthermore, compound **10** was stabilized by another hydrogen bond acceptor between the oxygen of acetamide moiety and the side chain of Tyr121 (distance: 2.95 Å). In compound **14**, a dual hydrogen bonding network was involved between the nitrogen atom and the NH proton of the phenylhydrazone moiety, and the side chains of Ser59 and Thr56, respectively (distance: 2.39 and 2.42 Å, respectively). The other oxygen of thioxopyrimidine-4,6-dione in compound **15** mediated a strong hydrogen bond acceptor with the side chains of Thr56 (distance: 2.49 Å) (Figure 5).

From the docking results, it is evident that the thioxopyrimidine-4,6-dione moiety linked to the 1,3,4-thiadiazole scaffold contributes to the activity of compounds **10**, **13**, **14**, and **15** through H-bonds to Ser59, which considerably strengthens the binding interaction (Figure 6).

## 3. Materials and Methods

### 3.1. Chemistry

All solvents, reagents, and chemicals were obtained from Alfa Aesar (Ward Hill, MA, USA) and Sigma-Aldrich (St. Louis, MO, USA). All melting points are not corrected and were measured on a Stuart SMP 30 advanced digital electric melting point apparatus (Cole-Parmer, Staffordshire, UK). Infrared spectra were recorded on a Shimadzu FT-IR 8300 E (Shimadzu Corporation, Kyoto, Japan), using KBr discs and are reported as ν cm^−1^. A Bruker Avance III spectrometer (Bruker Corporation, Rheinstetten, Germany) was used to record ^1^H-NMR spectra at 400 MHz using TMS as an internal standard and DMSO-d6 as a solvent. ^13^C-NMR spectra were recorded on the same spectrometer at 100 MHz using the same solvent. MS spectra were measured on a Shimadzu GC-MS-QP-1000 EX mass spectrometer instrument operating at 70 eV. The elemental analyses of the new compounds were recorded on a Perkin-Elmer CHN-2400 analyzer (Waltham, MA, USA) and carried out at the Microanalytical Centre, Cairo University, Cairo, Egypt. The microanalysis showed that the observed values were within ±0.4% of theoretical values. The homogeneity of the compounds and the progress of the chemical reactions were monitored by TLC silica gel plates (60F_254_; Merck, Munchen, Germany). The biological evaluation was conducted at the Department of Pharmacology, Faculty of Pharmacy, Mansoura University, Egypt. Compound **1** [40] was prepared using a previously reported method [41] (m.p. 217–219 °C, Let. 215 °C).

#### 3.1.1. *N*-(5-(3,5-Dinitrophenyl)-1,3,4-thiadiazol-2-yl)hydrazinecarbothioamide (**2**)

To a solution of compound **1** (2.67 g, 0.01 mol), in THF (10 mL), potassium hydroxide (2.5 g) in tetrahydrofuran (10 mL) was added portion-wise with shaking. To the reaction mixture, carbon disulfide (1.2 mL, 0.02 mol) was added with stirring for 1 h in an ice bath at 5 °C, followed by the addition of hydrazine hydrate (0.98 mL, 0.02 mol) with stirring for 1 h at 60 °C. The reaction mixture was concentrated under a vacuum, and the obtained solid was filtered off and crystalized from dioxane to produce the thiosemicarbazide derivative **2**. Yield: 87%, m.p: 260–262 °C, IR (KBr, cm^−1^): ν = 3430–3273 (NH_2_, NH), 3083 (C‒H_arom_), 1629 (C=N). ^1^H-NMR (400 MHz, ppm, DMSO-d_6_): *δ* = 5.33 (s, 2H, NH_2_, D_2_O exchangeable), 8.79, 9.13, 9.31 (3s, 3H, Ar-H), 11.11, 11.67 (2s, 2H, 2 NH, D2O exchangeable). ^13^C-NMR (100 MHz, ppm, DMSO-d_6_): *δ* = 125.3, 127.6, 133.4, 142.5, 147.9, 150.6, 184.2 (9 C). MS (EI, 70 eV): *m*/*z* (%) *=* 341 [M^+^, 13]. Analysis for C_9_H_7_N_7_O_4_S_2_ (341.32): Calcd. C, 31.67; H, 2.07; N, 28.73. Found: C, 32.01; H, 1.89; N, 29.08.

#### 3.1.2. 5-(1-Amino-2-phenylethyl)-*N*-(5-(3,5-dinitrophenyl)-1,3,4-thiadiazol-2-yl)-1,3,4-thiadiazol-2-amine (**3**)

A mixture of the thiosemicarbazide derivative **2** (3.41 g, 0.01 mol) and phenylalanine (1.65 g, 0.01 mol) in pyridine (15 mL) was heated in an oil bath at 180–200 °C for 6 h. After cooling, the reaction mixture was treated with a cold Na_2_CO_3_ solution (10%). The formed solid was filtered off, washed with H_2_O, and crystallized from ethanol to give **3**. Yield: 51%, m.p.: 238–240 °C, IR (KBr, cm^−1^): ν = 3415–3257 (NH_2_, NH), 3058 (C‒H_arom_), 2958 (C‒H_aliph_), 1630 (C=N). ^1^H-NMR (400 MHz, ppm, DMSO-d_6_): *δ* = 3.17 (d, 2H, CH_2_-Ph), 4.08 (t, 1H, *J* = 6.8 Hz, CH-N), 6.45 (s, 2H, NH2, D2O exchangeable), 7.48–7.71 (m, 5H, Ph-H), 8.68, 9.01, 9.26 (3s, 3H, Ph-H), 11.21 (s, 1H, NH, D2O exchangeable). ^13^C-NMR (100 MHz, ppm, DMSO-d_6_): *δ* = 37.9, 55.1, 125.4, 126.1, 128.2, 128.8, 129.9, 130.2, 132.7, 136.3, 143.2, 147.1, 150.3, 156.9 (18 C). MS (EI, 70 eV): *m*/*z* (%) *=* 470 [M^+^, 9]. Analysis for C_18_H_14_N_8_O_4_S_2_ (470.48): Calcd. C, 45.95; H, 3.00; N, 23.82. Found: C, 46.23; H, 3.29; N, 24.19.

#### 3.1.3. 5-(3,5-Dinitrophenyl)-*N*-(5-phenyl-1,3,4-thiadiazol-2-yl)-1,3,4-thiadiazol-2-amine (**4**)

To a mixture of **2** (3.41 g, 0.01 mol), benzoic acid (1.22 g, 0.01 mol) and phosphorous oxychloride (5 mL) were added and refluxed in a water bath for 45 min, then cooled and quenched with cold water (10 mL). The resulting solution was refluxed for an additional 4 h and filtered while hot. The filtrate was cooled and triturated with an aqueous potassium hydroxide solution. The obtained solid was separated by filtration, dried, and crystallized from dioxin to produce compound **4**. Yield 70%, m.p. 277–279 °C, IR (KBr, cm^−1^): ν = 3265 (NH), 3033 (CH_arom_), 1616 (C=N). ^1^H-NMR (400 MHz, ppm, DMSO-d_6_): *δ* = 7.33–7.66 (m, 5H, Ph-H), 8.64, 9.07, 9.22 (3s, 3H, Ph-H), 10.22 (s, 1H, NH, D2O exchangeable). ^13^C-NMR (100 MHz, ppm, DMSO-d_6_): *δ* = 124.5, 126.8, 127.5, 128.4, 129.8, 130.4, 131.3, 137.2, 143.2, 147.7, 151.1, 157.8 (16 C). MS (EI, 70 eV): *m*/*z* (%) *=* 427 [M^+^, 24]. Analysis for C_16_H_9_N_7_O_4_S_2_ (427.41): Calcd. C, 44.96; H, 2.12; N, 22.94. Found: C, 45.29; H, 2.00; N, 23.28.

#### 3.1.4. 5-{[5-(3,5-Dinitrophenyl)-1,3,4-thiadiazol-2-yl]amino}-1,3,4-thiadiazole-2-thiol (**5**)

To a solution of thiosemicarbazide **2** (3.41 g, 0.01 mol) in ethanolic sodium hydroxide (20 mL, 2%), carbon disulfide (0.9 mL, 0.015 mol) was added with stirring for 30 min. The reaction mixture was refluxed for 12 h, and, after cooling, acidified with hydrochloric acid. The separated solid was filtered off, washed with water, and crystallized from dioxane to give compound **5**. Yield 62%, m.p. 286–288 °C, IR (KBr, cm^−1^): ν = 3261 (NH), 3044 (C‒H_arom_), 1622 (C=N). ^1^H-NMR (400 MHz, ppm, DMSO-d_6_): *δ* = 8.71, 9.11, 9.29 (3s, 3H, Ar-H), 10.13 (s, 1H, NH, D_2_O exchangeable), 12.23 (s, 1H, NH, D_2_O exchangeable). ^13^C-NMR (100 MHz, ppm, DMSO-d_6_): *δ* = 126.8, 128.4, 129.8, 131.3, 147.7, 151.1, 157.8, 181.5 (10 C). MS (EI, 70 eV): *m*/*z* (%) *=* 383 [M^+^, 20]. Analysis for C10H5N7O4S3 (383.38): Calcd. C, 31.33; H, 1.31; N, 25.58. Found: C, 30.98; H, 1.52; N, 25.95.

#### 3.1.5. 2-((5-((5-(3,5-Dinitrophenyl)-1,3,4-thiadiazol-2-yl)amino)-1,3,4-thiadiazol-2-yl)thio)-1-phenylethan-1-one (**6**)

To equimolar amount of **5** (3.83 g, 0.01 mol) and phenacyl bromide (1.99 g, 0.01 mol) were dissolved in dry acetone (30 mL), potassium carbonate anhydrous (1.38 g, 0.01 mol) was added, followed by refluxing on a water bath for 10 h. The reaction mixture was filtered and the filtrate was poured over cooled water; the obtained solid was filtered off and crystallized from benzene to produce compound **6**. Yield 62%, m.p. 230–232 °C, IR (KBr, cm^−1^): ν = 3269 (NH), 3057 (C‒H_arom_), 2921 (C‒H_aliph_), 1618 (C=N). ^1^H-NMR (400 MHz, ppm, DMSO-d_6_): *δ* = 4.96 (s, 2H, S-CH2-CO), 7.37–7.69 (m, 5H, Ph-H), 8.75, 9.17, 9.24 (3s, 3H, Ar-H), 10.29 (s, 1H, NH, D_2_O exchangeable). ^13^C-NMR (100 MHz, ppm, DMSO-d_6_): *δ* = 40.5, 126.2, 127.5, 128.7, 129.1, 129.7, 130.7, 131.5, 132.3, 146.9, 151.16, 152.2, 156.9, 179.6 (18 C). MS (EI, 70 eV): *m*/*z* (%) *=* 501 [M^+^, 32]. Analysis for C_18_H_11_N_7_O_5_S_3_ (501.51): Calcd. C, 43.11; H, 2.21; N, 19.55. Found: C, 42.80; H, 2.00; N, 19.21.

#### 3.1.6. 5-(5-((5-(3,5-Dinitrophenyl)-1,3,4-thiadiazol-2-yl)amino)-1,3,4-thiadiazol-2(3H)-ylidene)pyrimidine-2,4,6(1H,3H,5H)-trione (**8**)

To a solution of compound **2** (3.41 g, 0.01 mol) in methanol (30 mL), 5-[bis(methylthio)- methylene] barbituric acid (**7**) (2.32 g, 0.01 mol) was added with stirring and refluxed for 5 h. The reaction mixture was left to cool and the separated precipitate was filtered off and recrystallized from dioxane to give compound **8**. Yield 67%, m.p. 293–295 °C, IR (KBr, cm^−1^): ν = 3322–3189 (NH), 1744, 1678, 1655 (3 C=O). ^1^H-NMR (400 MHz, ppm, DMSO-d_6_): *δ* = 8.70, 9.14, 9.30 (3s, 3H, Ph-H), 9.34, 11.19, 12.67, 12.78 (4s, 4H, 4NH, D_2_O exchangeable). ^13^C-NMR (100 MHz, ppm, DMSO-d_6_): *δ* = 113.7, 127.5, 128.2, 130.7, 132.3, 146.4, 148.1, 154.8, 157.2, 162.6, 167.2, 167.8 (14 C). MS (EI, 70 eV): *m*/*z* (%) *=* 477 [M^+^, 23]. Analysis for C_14_H_7_N_9_O_7_S_2_ (477.39): Calcd. C, 35.22; H, 1.48; N, 26.41. Found: C, 34.88; H, 1.70; N, 26.81.

#### 3.1.7. 3-[5-(3,5-Dinitrophenyl)-1,3,4-thiadiazol-2-yl]-2-hydrazonothiazolidin-4-one (**9**)

A mixture of thiosemicarbazide **2** (3.41 g, 0.01 mol) and chloroacetic acid (1 mL, 0.01 mol), sodium acetate (2.46 g, 0.03 mol) in acetic acid (20 mL) was refluxed for 6 h, cooled, and poured on water/ice. The obtained solid was filtered off, washed with water, and recrystallized from acetic acid to produce thiazolidin-4-one derivative **9**. Yield 78%, m.p. 246–248 °C, IR (KBr, cm^−1^): ν = 3378, 3254 (NH_2_), 2909 (C‒H_aliph_), 1681 (C=O), 1623 (C=N). ^1^H-NMR (400 MHz, ppm, DMSO-d_6_): *δ* = 3.86 (s, 2H, CH_2_, thiazolidinone), 6.41 (s, 2H, NH_2_, D_2_O exchangeable), 8.77, 9.17, 9.28 (3s, 3H, Ph-H). ^13^C-NMR (100 MHz, ppm, DMSO-d_6_): *δ* = 31.2, 126.9, 129.6, 131.5, 133.8, 147.5, 150.4, 155.2, 177.7 (11 C). MS (EI, 70 eV): *m*/*z* (%) *=* 381 [M^+^, 20]. Analysis for C_11_H_7_N_7_O_5_S_2_ (381.34): Calcd. C, 34.65; H, 1.85; N, 25.71. Found: C, 35.01; H, 2.12; N, 26.03.

#### 3.1.8. *N*-Acetyl-*N*-[3-(5-(3,5-dinitrophenyl)-1,3,4-thiadiazol-2-yl]-4,6-dioxo-2-thioxotetrahydro-pyrimidin-1(2H)-yl)acetamide (**10**)

A mixture of **2** (3.41 g, 0.01 mol), malonic acid (1.04 g, 0.01 mol) and acetyl chloride (8 mL) was refluxed for 10 h. The mixture was poured into cold water with stirring; the precipitated solid was filtered off and crystallized from dioxane to give compound **10**. Yield 59%, m.p. 271–273 °C, IR (KBr, cm^−1^): ν = 2944 (C‒H_aliph_), 1710, 1691, 1661 (3 C=O), 1616 (C=N). ^1^H-NMR (400 MHz, ppm, DMSO-d_6_): *δ* = 2.72 (s, 6H, 2CH_3_), 3.23 (s, 2H, CH_2_, pyrimidine), 8.73, 9.14, 9.32 (3s, 3H, Ph-H). ^13^C-NMR (100 MHz, ppm, DMSO-d_6_): *δ* = 24.1, 41.9, 127.5, 129.9, 132.5, 137.6, 148.1, 151.3, 163.5, 164.8, 168.5, 175.9 (16 C). MS (EI, 70 eV): *m*/*z* (%) *=* 493 [M^+^, 18]. Analysis for C_16_H_11_N_7_O_8_S_2_ (493.43): Calcd. C, 38.95; H, 2.25; N, 19.87. Found: C, 39.31; H, 2.00; N, 20.20.

#### 3.1.9. 1-[5-(3,5-Dinitrophenyl)-1,3,4-thiadiazol-2-yl]-3-phenylthiourea (**11**)

To aminothiazole derivative **1** (2.67 g, 0.01 mol) in dry dioxane (30 mL) and phenylisothio- cyanate (0.01 mol, 1.35 g), anhydrous potassium carbonate (1.38 g, 0.01 mol) was added with stirring and refluxed for 12 h, cold to room temperature then poured over ice/water. The obtained solid was filtered off, dried, and crystallized from dioxane to produce **11**. Yield 81%, m.p. 264–266 °C, IR (KBr, cm^−1^): ν = 3329, 3256 (2NH), 3080 (C‒H_arom_.), 1626 (C=N). ^1^H-NMR (400 MHz, ppm, DMSO-d_6_): *δ* = 7.45–7.89 (m, 5H, Ph-H), 8.68, 9.18, 9.29 (3s, 3H, Ph-H), 10.49, 11.33 (2s, 2H, 2NH, D_2_O exchangeable). ^13^C-NMR (100 MHz, ppm, DMSO-d_6_): *δ* = 127.3, 129.5, 131.6, 132.5, 134.1, 137.8, 147.7, 150.9, 154.5, 159.2, 182.9 (15 C). MS (EI, 70 eV): *m*/*z* (%) *=* 402 [M^+^, 16]. Analysis for C_15_H_10_N_6_O_4_S_2_ (402.40): Calcd. C, 44.77; H, 2.50; N, 20.89 Found: C, 45.08; H, 2.77; N, 21.21.

#### 3.1.10. *N*-[5-(3,5-Dinitrophenyl)-1,3,4-thiadiazol-2-yl]-3,4-diphenylthiazol-2(3H)-imine (**12**)

A mixture of **11** (4.02 g, 0.01 mol) and phenacyl bromide (1.99 g, 0.01 mol) in EtOH (50 mL), in the presence of TEA (0.5 mL), was refluxed for 10 h, then cooled to r.t. The formed solid was filtered off, washed with water, dried, and crystallized from ethanol to produce imine derivative **12**. Yield 51%, m.p. 251–253 °C, IR (KBr, cm^−1^): ν = 3061 (C‒H_arom_.), 1629 (C=N). ^1^H-NMR (400 MHz, ppm, DMSO-d_6_): *δ* = 6.65 (s, 1H, CH, thiazole), 7.41–7.91 (m, 10H, 2 Ph-H), 8.74, 9.12, 9.35 (3s, 3H, Ph-H). ^13^C-NMR (100 MHz, ppm, DMSO-d_6_): *δ* = 115.4, 122.4, 125.7, 126.8, 127.7, 129.9, 130.8, 131.4, 133.2, 133.9, 136.4, 137.6, 143.2, 147.9, 152.6, 155.2, 159.5 (23 C). MS (EI, 70 eV): *m*/*z* (%) *=* 502 [M^+^, 25]. Analysis for C_23_H_14_N_6_O_4_S_2_ (502.52): Calcd. C, 54.97; H, 2.81; N, 16.72. Found: C, 55.30; H, 3.00; N, 17.04.

#### 3.1.11. 1-[5-(3,5-Dinitrophenyl)-1,3,4-thiadiazol-2-yl]-3-phenyl-2-thioxodihydropyrimidine-4,6(1H,5H)-dione (**13**)

To a mixture of equimolar amounts of phenylthiourea derivative **11** (4.02 g, 0.01 mol) and malonic acid (1.04 g, 0.01 mol), acetyl chloride (5 mL) was added. The reaction mixture was heated in an oil bath at 140–150 °C for 4 h, then poured onto crushed ice/water. The reaction mixture was alkaloid with NaOH (pH = 9–10). The resulting solid was filtered off, washed with water, and crystallized from dioxane to give compound **13**. Yield 66%, m.p. 282–284 °C, IR (KBr, cm^−1^): ν = 3037 (C‒H_arom_.), 2961 (C‒H_aliph_), 1702, 1688 (C=O), 1624 (C=N). ^1^H-NMR (400 MHz, ppm, DMSO-d_6_): *δ* = 3.31 (s, 2H, CH_2_, pyrimidine) and 7.32–7.88 (m, 5H, Ph-H), 8.78, 9.10, 9.30 (3s, 3H, Ph-H). ^13^C-NMR (100 MHz, ppm, DMSO-d_6_): *δ* = 42.4, 126.3, 127.8, 129.5, 130.3, 131.4, 136.7, 143.2, 147.3, 153.1, 155.7, 169.2, 169.9, 187.2 (18 C). MS (EI, 70 eV): *m*/*z* (%) = 470 [M^+^, 20]. Analysis for C_18_H_10_N_6_O_6_S_2_ (470.43): Calcd. C, 45.96; H, 2.14; N, 17.86. Found: C, 45.61; H, 1.98; N, 18.20.

#### 3.1.12. 1-[5-(3,5-Dinitrophenyl)-1,3,4-thiadiazol-2-yl]-3-phenyl-5-(2-phenylhydrazono)-2-thioxo-dihydro-pyrimidine-4,6(1H,5H)-dione (**14**)

Benzene diazonium chloride (0.01 mol) [prepared by the addition of NaNO_2_ (0.01 mol) to distilled aniline (0.01 mol) in concentrated HCl (4 mL) at 0–5 °C under stirring] was added dropwise to a solution of thiobarbituric derivative **13** (4.7 g, 0.01 mol) in ethanol (40 mL) containing sodium acetate (2 g), with stirring at 0–5 °C for 3 h. The reaction mixture was left at room temperature for 2 h; the solid product was filtrated off, washed with water and crystallized from dioxane to give hydrazone derivative **14**. Yield 58%, m.p. 267–269 °C, IR (KBr, cm^−1^): ν = 3276 (NH), 3029 (C‒H_arom_.), 1700, 1690 (C=O), 1620 (C=N). ^1^H-NMR (400 MHz, ppm, DMSO-d_6_): *δ* = 7.25–7.69 (m, 10H, 2Ph-H), 8.73, 9.14, 9.22 (3s, 3H, Ph-H), 12.55 (s, 1H, NH, D_2_O exchangeable). ^13^C-NMR (100 MHz, ppm, DMSO-d_6_): *δ* = 124.2, 126.5, 127.8, 129.9, 130.7, 131.1,131.8, 132.3, 133.4, 136.1, 139.3, 144.2, 146.8, 152.8, 154.8, 167.3, 168.5, 180.4 (24 C). MS (EI, 70 eV): *m*/*z* (%) *=* 558 [M^+^, 26]. Analysis for C_24_H_14_N_8_O_6_S_2_ (574.55): Calcd. C, 50.17; H, 2.46; N, 19.50. Found: C, 49.80; H, 2.64; N, 19.18.

#### 3.1.13. 5-Benzylidene-1-[5-(3,5-dinitrophenyl)-1,3,4-thiadiazol-2-yl]-3-phenyl-2-thioxodihydro-pyrimidine-4,6(1H,5H)-dione (**15**)

A mixture of **13** (4.7 g, 0.01 mol) and benzaldehyde (1.06 g, 0.01 mol) in EtOH (40 mL), containing piperidine (0.5 mL) as a catalyst, was heated under reflux for 8 h. The obtained solid was collected by filtration and crystallized from dioxane to give **15**. Yield 58%, m.p. 257–259 °C, IR (KBr, cm^−1^): ν = 3055 (C‒H_arom_.), 1698, 1682 (2 C=O), 1627 (C=N). ^1^H-NMR (400 MHz, ppm, DMSO-d_6_): *δ* = 6.84 (s, 1H, CH), 7.41–7.77 (m, 10H, 2Ph-H), 8.75, 9.08, 9.28 (3s, 3H, Ph-H). ^13^C-NMR (100 MHz, ppm, DMSO-d_6_): *δ* = 121.1, 125.3, 126.2, 127.6, 129.3, 130.1, 131.5, 132.8, 134.2, 137.3, 140.7, 144.4, 146.2, 150.6, 153.2, 155.4, 168.1, 169.2, 181.5 (25 C). MS (EI, 70 eV): *m*/*z* (%) *=* 574 [M^+^, 27]. Analysis for C_25_H_14_N_6_O_6_S_2_ (558.54): Calcd. C, 53.76; H, 2.53; N, 15.05. Found: C, 53.40; H, 2.79; N, 14.71.

#### 3.1.14. Dimethyl (5-(3,5-dinitrophenyl)-1,3,4-thiadiazol-2-yl)carbonimidodithioate (**16**)

Aminothiadiazole derivative **1** (2.67 g, 0.01 mol) was stirred in DMF (30 mL), then sodium hydroxide (5 mL, 20 M), carbon disulfide (1.52 g, 0.02 mol), and methyl iodide (1.43 g, 0.01 mol) were added in sequence at intervals of 30 min and stirring continued for 6 h. The mixture was then poured into an ice/water mixture with vigorous stirring. The formed precipitate was filtered off, washed with water, dried, and recrystallized from dioxane to give **16**. Yield 79%, m.p. 211–213 °C, IR (KBr, cm^−1^): ν = 3026 (C‒H_arom_.), 2887 (C‒H_aliph_), 1620 (C=N), 1334 (C‒S). ^1^H-NMR (400 MHz, ppm, DMSO-d_6_): *δ* = 2.56 (s, 6H, 2 CH_3_), 8.66, 9.13, 9.26 (3s, 3H, Ph-H). ^13^C-NMR (100 MHz, ppm, DMSO-d_6_): *δ* = 13.3, 126.5, 129.6, 132.8, 151.3, 153.8, 157.4, 168.1 (11 C). MS (EI, 70 eV): *m*/*z* (%) *=* 371 [M^+^, 14]. Analysis for C_11_H_9_N_5_O_4_S_3_ (371.40): Calcd. C, 35.57; H, 2.44; N, 18.86. Found: C, 35.21; H, 2.71; N, 19.22.

#### 3.1.15. Synthesis of *N*-[5-(3,5-dinitrophenyl)-1,3,4-thiadiazol-2-yl]benzo[d]azole derivatives **17**–**19**

A solution of **16** (3.71 g, 0.01 mol) in 15 mL *N*,*N*-dimethyl formamide was added to a solution of amino derivatives (namely, o-aminophenol, o-aminothiophenol, o-phenylenediamine, 0.01 mol) in 15 mL *N*,*N*-dimethyl formamide with stirring at room temperature for 30 min. The reaction mixture was refluxed for 8–10 h, left to cool, then poured on a water/ice mixture with stirring. The resulting solid was filtered and recrystallized from a solvent to produce compounds **17**–**19**, respectively.

#### 3.1.16. *N*-[5-(3,5-Dinitrophenyl)-1,3,4-thiadiazol-2-yl]benzo[*d*]oxazol-2-amine (**17**)

Yield 61%, m.p. 254–256 °C (EtOH), IR (KBr, cm^−1^): ν = 3389–3272 (NH), 3044 (C‒H_arom_.) and 1627 (C=N). ^1^H-NMR (400 MHz, ppm, DMSO-d_6_): *δ* = 7.17–7.51 (m, 4H, Ph-H), 8.62, 9.18, 9.32 (3s, 3H, Ph-H), 9.78 (s, 1H, NH, D_2_O exchangeable). ^13^C-NMR (100 MHz, ppm, DMSO-d_6_): *δ* = 120.2, 122.3, 125.6, 127.9, 130.5, 132.8, 138.1, 144.9, 146.6, 150.6, 154.1, 155.9, 157.4 (15 C). MS (EI, 70 eV): *m*/*z* (%) *=* 384 [M^+^, 16]. Analysis for C_15_H_8_N6O_5_S (384.33): Calcd. C, 46.88; H, 2.10; N, 21.87. Found: C, 46.55; H, 2.32; N, 22.20.

#### 3.1.17. *N*-[5-(3,5-Dinitrophenyl)-1,3,4-thiadiazol-2-yl]benzo[*d*]thiazol-2-amine (**18**)

Yield 68%, m.p. 281–283 °C (Dioxane), IR (KBr, cm^−1^): ν = 3364 (NH), 3039 (C‒H_arom_.), 1631 (C=N). ^1^H-NMR (400 MHz, ppm, DMSO-d_6_): *δ* = 7.36–7.67 (m, 4H, Ph-H), 8.69, 9.22, 9.33 (3s, 3H, Ph-H), 11.06 (s, 1H, NH, D_2_O exchangeable). ^13^C-NMR (100 MHz, ppm, DMSO-d_6_): *δ* = 119.6, 122.9, 126.1, 127.5, 131.7, 132.8, 137.3, 139.9, 145.8, 151.1, 154.5, 156.3, 158.8 (15 C). MS (EI, 70 eV): *m*/*z* (%) = 400 [M^+^, 16]. Analysis for C_15_H_8_N_6_O_4_S_2_ (400.39): Calcd. C, 45.00; H, 2.01; N, 20.99. Found: C, 44.68; H, 2.29; N, 21.34.

#### 3.1.18. *N*-(1H-Benzo[d]imidazol-2-yl)-5-(3,5-dinitrophenyl)-1,3,4-thiadiazol-2-amine (**19**)

Yield 63%, m.p. 274–276 °C (DMF). IR (KBr, cm^−1^): ν = 3355, 3282 (NH), 3052 (C‒H_arom_.), 1628 (C=N). ^1^H-NMR (400 MHz, ppm, DMSO-d_6_): *δ* = 7.11–7.43 (m, 4H, Ph-H), 8.74, 9.14, 9.27 (3s, 3H, Ph-H), 9.96 (s, 1H, NH, D_2_O exchangeable), 11.78 (s, 1H, NH, D_2_O exchangeable). ^13^C-NMR (100 MHz, ppm, DMSO-d_6_): *δ* = 120.9, 121.7, 122.4, 127.1, 128.1, 131.2, 138.1, 138.9, 146.3, 151.1, 155.2, 157.5, 159.4 (15 C). MS (EI, 70 eV): *m*/*z* (%) = 383 [M^+^, 19]. Analysis for C_15_H_9_N_7_O_4_S (383.34): Calcd. C, 47.00; H, 2.37; N, 25.58. Found: C, 46.70; H, 2.18; N, 25.25.

#### 3.1.19. *N*-(4,5-Dihydro-1H-imidazol-2-yl)-5-(3,5-dinitrophenyl)-1,3,4-thiadiazol-2-amine (**20**)

To a solution of dimethyl carbonimidodithioate derivative **16** (3.71 g, 0.01 mol) in dimethyl- formamide (15 mL), ethylene diamine (1.2 g, 0.02 mol), in DMF (10 mL) was added drop wise with stirring at room temperature. The reaction mixture was refluxed for 8 h, left to cool, then poured onto water/ice with stirring. The obtained solid was filtered off, washed with water, dried, and recrystallized from ethanol to produce compound **20**. Yield 55%, m.p. 227–229 °C, IR (KBr, cm^−1^): ν = 3371, 3272 (NH), 3049 (C‒H_arom_.), 1622 (C=N). ^1^H-NMR (400 MHz, ppm, DMSO-d_6_): *δ* = 2.69 (t, 2H, *J* = 8.2 Hz, CH_2_-imidazole), 2.81 (t, 2H, *J* = 8.2 Hz, CH_2_-imidazole), 8.70, 9.10, 9.25 (3s, 3H, Ph-H), 9.83 (s, 1H, NH, D_2_O exchangeable), 10.66 (s, 1H, NH, D_2_O exchangeable). ^13^C-NMR (100 MHz, ppm, DMSO-d_6_): *δ* = 46.9, 47.5, 121.7, 127.5, 131.8, 146.9, 153.8, 154.6, 159.4 (11 C). MS (EI, 70 eV): *m*/*z* (%) = 335 [M^+^, 12]. Analysis for C_11_H_9_N_7_O_4_S (335.30): Calcd. C, 39.40; H, 2.71; N, 29.24. Found: C, 39.74; H, 3.00; N, 24.95.

### 3.2. Biological Evaluation

#### 3.2.1. Antimicrobial Sensitivity Assay

The antimicrobial activities of the synthesized compounds were evaluated against four bacterial strains, *Staphylococcus aureus*, *Bacillus subtilis*, *Pseudomonas aeruginosa*, and *Escherichia coli*, and two fungal strains, *Aspergillus niger* and *Candida albicans*, using a standard well agar diffusion assay according to Cheesbrough et al. [35]. Plates containing nutrient agar medium and sabouraud dextrose agar medium (for bacteria and fungi, respectively) were surface-inoculated with 106 CFU/mL of freshly prepared microorganisms. Using a 6-mm sterile cork borer, wells were punched in the agar and filled separately with 100 µL of the tested compounds (100 µg/mL in DMSO). The plates were left in a refrigerator for 2 h to allow diffusion of the tested compounds. After that, the plates were incubated for 24 h at 37 °C for bacteria and for 72 h at 28 °C for fungi, then the inhibition zones surrounding the wells were measured in millimeters. Amoxicillin and Fluconazole were used as the standard against bacteria and fungi, respectively, using the same concentration (100 μg/mL).

#### 3.2.2. In Vitro Anticancer Activity

The newly synthesized heterocyclic compounds were evaluated for their in vitro cytotoxicity against four cancer cell lines, CCRF-CEM (leukemia), HCT-15 (human colon carcinoma), PC-3 (prostate cancer) and UACC-257 (melanoma, skin cancer) cell lines, which were obtained from Sigma-Aldrich Chemical Company, USA. DOX (Doxorubicin) was utilized as a reference drug according to a previously reported MTT method [38,39].

#### 3.2.3. Dihydrofolate Reductase (DHFR) Inhibition

The in vitro DHFR enzyme inhibition assessment was carried out in a confirmatory diagnostic unit, Vacsera, Egypt. All synthesized derivatives **1**–**20** were screened against DHFR using Methotrexate as a reference according to a previously reported method [36]. The results are reported as IC50 values of enzymatic activity in Table 3.

### 3.3. Molecular Modeling Studies

The docking study was performed using Molecular Operating Environment (MOE^®^) 2008.10 software [38,39]. The X-ray crystal structure of the dihydro–folate reductase enzyme was downloaded from the Protein Data Bank website (PDB ID: 1DLS) [37]. Regularization and optimization for ligand and protein were done. The performance of the docking method was evaluated by re-docking the crystal ligand into the assigned active DHFR enzyme to evaluate a root-mean-square deviation value. Then, the molecular docking was applied for compounds **10**, **13**, **14**, and **15** into ATP binding site of DHFR according to the reported method [38,39].

## 4. Conclusions

In summary, a series of 1,3,4-thiadiazole derivatives **2**–**20** incorporating with different heterocyclic systems was designed and synthesized. All synthesized compounds were examined for their in vitro antimicrobial, antitumor, and DHFR inhibition activity. The antimicrobial results exhibited the ability of the compounds **9**, **10**, **13**, and **16** to inhibit the growth of a panel of six strains with higher inhibition zones in comparison with the reference drugs. In addition, the cytotoxic activity against four cell lines illustrated that compounds **6**, **14**, and **15** have mostly prevented cell growth with lower IC_50_ values. Based on the data obtained from the DHFR inhibition study, compounds **10**, **13**, **14**, and **15**, containing a thioxopyrimidine-4,6-dione moiety at p-2 of the parent 1,3,4-thiadiazole ring, were the most potent derivatives in comparison with Methotrexate. Moreover, the docking study indicated that compounds **10**, **13**, **14**, and **15** showed good fitting and caused favorable contacts in the binding site of DHFR enzyme. Therefore, the obtained mark points have been proposed as an explanation for the unique activity of such derivatives and could be used as a template for further development and future optimization of new antimicrobial and anticancer agents via DHFR inhibition.

## Figures and Tables

**Table 1:** In vitro antimicrobial activities of the tested compounds **1**–**20** at 100 μg/mL and expressed as inhibition zone diameter (mm).

	Diameter of Inhibition Zone (mm)					
Compound Number	Gram Positive Bacteria		Gram Negative Bacteria		Fungi	
	*Staphylococcus aureus*	*Bacillus subtilis*	*Pseudomonas aeruginosa*	*Escherichia coli*	*Aspergillus niger*	*Candida albicans*
**1**	12	10	14	8	6	9
**2**	25	24	22	23	17	18
**3**	0	0	0	2	0	0
**4**	0	0	2	3	0	0
**5**	20	19	20	21	16	18
**6**	10	11	13	12	9	8
**8**	9	7	8	10	8	9
**9**	27	28	26	26	21	23
**10**	28	29	27	27	22	24
**11**	13	14	16	15	11	13
**12**	0	2	0	2	0	0
**13**	30	29	28	27	23	25
**14**	18	13	11	9	8	12
**15**	16	19	13	10	6	15
**16**	28	26	25	27	22	22
**17**	8	8	6	9	7	6
**18**	4	3	0	3	2	3
**19**	5	0	6	4	3	4
**20**	21	18	20	22	16	18
**Amoxicillin**	28	27	24	25	-	-
**Fluconazole**	-	-	-	-	20	22

**Table 2:** In vitro cytotoxic activity of the tested compounds **1**–**20** against different human cancer cell lines.

Compound Number	IC_50_ (Mean ± SEM) (µM) ^a^				
	CCRF-CEM	HCT-15	PC-3	UACC-257	MCF-10A
**DOX ^b^**	6.78 ± 0.7	5.17 ± 0.2	4.56 ± 0.4	7.34 ± 0.5	377.33 ± 2.55
**1**	90.7 ± 1.2	79.8 ± 2.6	88.2 ± 1.6	81.6 ± 1.1	497.54 ± 5.44
**2**	74.56 ± 3.8	67.20 ± 2.9	70.18 ± 3.2	71.20 ± 3.9	586.43 ± 4.53
**3**	51.88 ± 1.6	30.12 ± 1.8	27.24 ± 1.2	46.44 ± 1.1	464.34 ± 3.47
**4**	39.28 ± 2.6	32.75 ± 2.2	29.52 ± 1.5	36.33 ± 2.4	383.63 ± 2.56
**5**	77.74 ± 2.6	65.66 ± 3.3	82.55 ± 1.9	80.66 ± 2.2	252.74 ± 2.45
**6**	10.99 ± 0.3	9.87 ± 0.5	9.92 ± 1.2	12.67 ± 0.6	461.85 ± 5.58
**8**	19.19 ± 0.5	17.24 ± 0.9	15.44 ± 1.4	22.66 ± 1.3	373.96 ± 6.49
**9**	76.22 ± 2.6	69.65 ± 2.7	81.59 ± 1.3	83.33 ± 2.9	262.07 ± 4.50
**10**	40.44 ± 1.9	36.76 ± 1.8	33.88 ± 1.1	38.55 ± 2.1	351.89 ± 6.68
**11**	71.26 ± 3.2	63.27 ± 2.3	66.14 ± 3.6	73.34 ± 3.5	380.68 ± 5.57
**12**	15.78 ± 0.8	15.01 ± 0.5	12.14 ± 1.1	18.38 ± 1.6	373.47 ± 6.45
**13**	49.36 ± 1.2	52.72 ± 1.2	48.88 ± 2.1	55.55 ± 0.9	460.56 ± 8.64
**14**	9.66 ± 0.6	8.59 ± 0.7	6.99 ± 1.2	9.20 ± 0.8	474.44 ± 8.72
**15**	6.99 ± 0.4	5.28 ± 0.5	4.67 ± 0.3	7.41 ± 0.5	352.35 ± 7.53
**16**	79.32 ± 3.1	68.33 ± 4.3	80.59 ± 1.7	76.61 ± 1.7	473.26 ± 8.44
**17**	67.45 ± 3.4	61.27 ± 2.3	62.56 ± 2.6	70.77 ± 1.5	382.35 ± 7.35
**18**	58.58 ± 4.4	55.67 ± 1.6	61.21 ± 1.1	65.34 ± 2.2	491.24 ± 9.53
**19**	84.44 ± 2.2	69.87 ± 3.3	86.95 ± 1.5	83.54 ± 2.9	382.32 ± 8.72
**20**	83.34 ± 4.2	71.64 ± 4.1	92.34 ± 4.2	88.64 ± 4.1	473.23 ± 9.83

^a^ IC_50_, compound concentration required to inhibit tumor cell proliferation by 50%, ^b^ positive control, SEM = standard error mean; each value is the mean of three values.

**Table 3:** In vitro inhibitory activities of the tested compounds **1**–**20** against DHFR enzyme.

Compound Number	IC_50_ (Mean ± SEM) (µM)
	DHFR
**1**	17.26 ± 0.43
**2**	14.33 ± 0.81
**3**	28.54 ± 0.22
**4**	31.57 ± 0.30
**5**	15.38 ± 0.12
**6**	13.45 ± 0.23
**8**	20.54 ± 0.14
**9**	8.46 ± 0.13
**10**	1.00 ± 0.85
**11**	17.13 ± 0.90
**12**	36.48 ± 0.72
**13**	0.09 ± 0.91
**14**	0.08 ± 0.37
**15**	0.04 ± 0.82
**16**	10.24 ± 0.97
**17**	27.42 ± 0.35
**18**	24.71 ± 1.26
**19**	29.00 ± 1.25
**20**	11.80 ± 0.79
**Methotrexate**	0.14 ± 1.38

IC_50_: Compound concentration required to inhibit DHFR enzyme activity by 50%, SEM = standard error mean; each value is the mean of three values.
